# Three new species, one new genus and subfamily of Dorylaimida (de Man, 1876) Pearse, 1942, and revisions on the families Tylencholaimellidae Jairajpuri, 1964 and Mydonomidae Thorne, 1964 (Nematoda: Dorylaimida)

**DOI:** 10.7717/peerj.7541

**Published:** 2019-08-26

**Authors:** Wen-Jia Wu, Chun-Ling Xu, Hui Xie, Dong-Wei Wang

**Affiliations:** 1 College of Agriculture, South China Agricultural University, Lab of Plant Nematology/Research Center of Nematodes of Plant Quarantine, Department of Plant Pathology/Guangdong Province Key Laboratory of Microbial Signals and Disease Control, Guangzhou, Guangdong, China; 2 South China Botanical Garden, Chinese Academy of Sciences, Key Laboratory of Vegetation Restoration and Management of Degraded Ecosystems, Guangzhou, Guangdong, China

**Keywords:** *Paratylencholaimus sanshaensis* gen. nov. sp. nov., *Tylencholaimus zhongshanensis* sp. nov., *Dorylaimoides shapotouensis* sp. nov., *Paratylencholaiminae* n. subfam., Key, Phylogenetic analysis, Revisions, Tylencholaimelliae, Mydonomidae

## Abstract

Three new species of the order Dorylaimida (de Man, 1876) Pearse, 1942 were identified and described. *Paratylencholaimus sanshaensis* gen. nov. sp. nov. from Hainan is proposed as a new member of the family Tylencholaimellidae Jairajpuri, 1964. *Paratylencholaimus* gen. nov. is close to *Phellonema* Thorne, 1964 and *Goferus* Jairajpuri & Ahmad, 1992 but can be differentiated mainly by having basal part of odontophore rod-like and without knobs, and basal part of pharynx expanded gradually. *Tylencholaimus zhongshanensis* sp. nov. from Guangdong and *Dorylaimoides shapotouensis* sp. nov. from the Ningxia Hui Autonomous Region are also described herein. Phylogenetic analyses based on the 18S rDNA and the D2–D3 region of the 28S rDNA support that the three new species are valid. The classifications of the families Tylencholaimellidae and Mydonomidae Thorne, 1964 are revised mainly based on the analysis of the morphology of odontostyle and odontophore. After these revisions, Paratylencholaiminae subfam. nov. including *Paratylencholaimus* gen. nov. and *Goferus* is proposed. *Athernema* and *Agmodorus* of Tylencholaimellidae are transferred into Mydonomidae, and the subfamily Athernematinae of Tylencholaimellidae is dismissed. The main characteristics of the family Mydonomidae and Tylencholaimellidae are revised. Keys to the genera of Mydomonidae and Tylencholaimellidae are included.

## Introduction

In the classification proposed by Andrássy (2009), which is based on the classification created by Peña Santiago (2006), the superfamily Tylencholaimoidea contains a wide range of genera and species and includes six families: Leptonchidae Thorne, 1935, Tylencholaimidae Filipjev, 1934, Mydonomidae Thorne, 1964, Tylencholaimellidae Jairajpuri, 1964, Aulolaimoididae Jairajpuri, 1964 and Encholaimidae Golden & Murphy, 1967. Tylencholaimoidea can be differentiated from other superfamilies of Dorylaimida (de Man, 1876) Pearse, 1942 mainly by having tylencholaimoid or dorylaimoid cuticle, cap-like lip region, markedly short basal expansion of the pharynx, common occurrence of both pro- and opisthodelphy, and few male supplements. However, Andrássy (2009) stated that this classification of Tylencholaimoidea should be artificial as the species of this superfamily can ‘hardly represent a homogeneous trend in the evolution of this group’. Peña Santiago (2014) again stressed that the superfamilies classification of the suborder Dorylaimina Pearse, 1936 is not supported by morphology or molecular analyses (Holterman et al., 2008). Peña Santiago (2014) canceled the superfamilies of Dorylaimina and kept the families, and moved Encholaimidae under Nordiidae.

During nematode investigations in China, three new species of Dorylaimina were identified. One from Guangdong belongs to the genus *Tylencholaimus* de Man, 1876 (Tylencholaimidae), and the second one from the Ningxia Hui Autonomous Region is a new member of the genus *Dorylaimoides* Thorne & Swanger, 1936 (Mydonomidae). These species are herein described as *Tylencholaimus zhongshanensis* sp. nov. and *Dorylaimoides shapotouensis* sp. nov. The third one from Hainan is interesting. It equips with dorylaimoid cuticle that is different from Tylencholaimidae (equips with tylencholaimoid cuticle), but other characteristics are highly similar to those of Tylencholaimidae. And later, three more populations of this species from Guangdong were collected. With further examinations, this species was suggested to be a member of a new genus of the family Tylencholaimellidae, herein described as *Paratylencholaimus sanshaensis* gen. nov. sp. nov. Detailed descriptions based on microscopy and phylogenetic analysis based on the 18S rDNA and the D2–D3 region of the 28S rDNA of the three new species were presented. In addition, the classification of Tylencholaimellidae and Mydonomidae was discussed, one new subfamily of Tylencholaimellidae was proposed, and keys to the genera of the revised Tylencholaimellidae and Mydonomidae were provided.

## Materials & Methods

### Morphology and morphometrics

Soil samples were collected from the rhizosphere soil of some plants from Hainan, Guangdong and Ningxia, respectively. Nematode populations were extracted from samples using the modified Baermann funnel method (Whitehead & Hemming, 1965). Then, specimens were gently killed at 62 °C for 3 min, fixed in 4% FG fixative, dehydrated using the glycerol-ethanol method and then mounted on permanent slides for further examination (Xie, 2005). The specimens were observed, measured and photographed as described by Wu et al. (2017). Locations of the pharyngeal gland nuclei were measured as described previously (Andrássy, 1998). Measurements are given as mean (minimum-maximum) with SD indicated when *n* > 30. Nematodes were prepared for SEM observations as described by Abolafia & Peña Santiago (2005) and observed with a FEI XL-30-ESEM electron microscope at 10 KV.

### DNA extraction, amplification and sequencing

A single nematode was placed into 10 µL mixed solution (distilled water: 2 ×buffer for KOD FX = 1:1) and cut using a sterilized needle. The genomic DNA was extracted by adding 1 µL 20 µg/mL proteinase K and then reacting at 65 °C for 1 h and 95 °C for 15 min. PCR reaction systems were performed in a 10 µL reaction mixture containing 5 µL of 2 ×buffer for KOD FX, 0.3 µL of each primer (10 µM), 2 µL of dNTPs (200 µM), 1 µL of DNA, 1.2 µL of distilled water and 0.2 µL of KOD FX (1 U/µL). Two overlapping fragments of the 18S rDNA were amplified using primer set 988F (5′–CTCAAAGATTAAGCCATGC–3′) and 1912R (5′–TTTACGGTCAGAACTAGGG–3′) for the first fragment, and 1813F (5′–CTGCGTGAGAGGTGAAAT–3′) and 2646R (5′–GCTACCTTGTTACGACTTTT–3′) for the second fragment (Holterman et al., 2006; Nedelchev et al., 2014). For the amplification of D2–D3 region of the 28S rDNA, the primer set D2A (5′–ACAAGTACCGTGAGGGAAAGTTG–3′) and D3B (5′–TCGGAAGGAACCAGCTACTA–3′) (De Ley et al., 1999) were used. The PCR reactions were performed as described previously (Wu et al., 2017). The newly obtained sequences of the new species were deposited in GenBank.

### Phylogenetic analysis

The sequences of the three new species were respectively compared with sequences in GenBank using BLAST. Sequences of species of Tylencholaimidae, Leptonchidae, Mydonomidae, Tylencholaimellidae, Mononchida Jairajpuri, 1969 and Nygolaimina Ahmad & Jairajpuri, 1979 were aligned along with the sequences of the three new species. The sequence alignments were performed, and conservative regions were selected using MEGA v6. For the Bayesian inference (BI) analysis, the substitution saturation was tested by DAMBE. The best-fit models were selected by AIC (Akaike Information Criterion) in MrModeltest v2.3. Bayesian trees were constructed by using MrBayes v3.1.2 running the chain for 1,000,000 generations with a sample frequency of 1,000 generations, and setting the ‘burnin’ at 2500. The topologies were used to generate a 50% majority rule consensus tree. Posterior probabilities (PP) were given for appropriate clades.

### Nomenclatural acts

The electronic version of this article in Portable Document Format (PDF) will represent a published work according to the International Commission on Zoological Nomenclature (ICZN), and hence the new names contained in the electronic version are effectively published under that Code from the electronic edition alone. This published work and the nomenclatural acts it contains have been registered in ZooBank, the online registration system for the ICZN. The ZooBank LSIDs (Life Science Identifiers) can be resolved and the associated information viewed through any standard web browser by appending the LSID to the prefix http://zoobank.org/. The LSID for this publication is: urn:lsid:zoobank.org:pub:427B5E52-23B0-4474-B179-4FA9FC5E7C9C. The online version of this work is archived and available from the following digital repositories: PeerJ, PubMed Central and CLOCKSS.

## Results

**Table:** 

**Figure 1:**
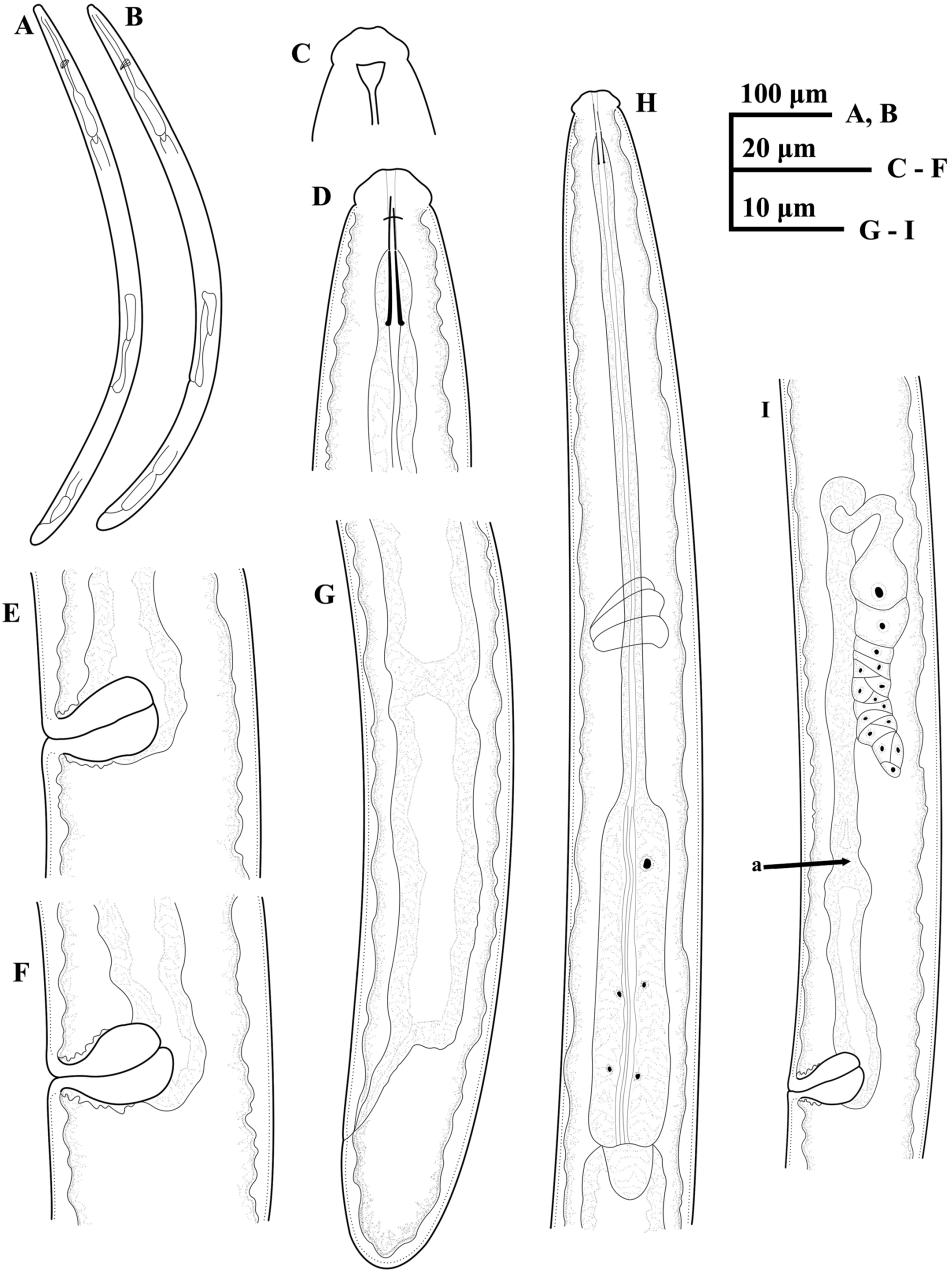
Ink drawing of *Tylencholaimus zhongshanensis* sp. nov. Female: (A, B) Entire body. (C) Amphidial fovea. (D) Anterior region. (E, F) Vulvas in lateral view. (G) Posterior region. (H) Pharynx. (I) Genital system (a: Boundary between the oviduct and uterus). Holotype: C. Paratypes: A, B, D–I.

**Figure 2:**
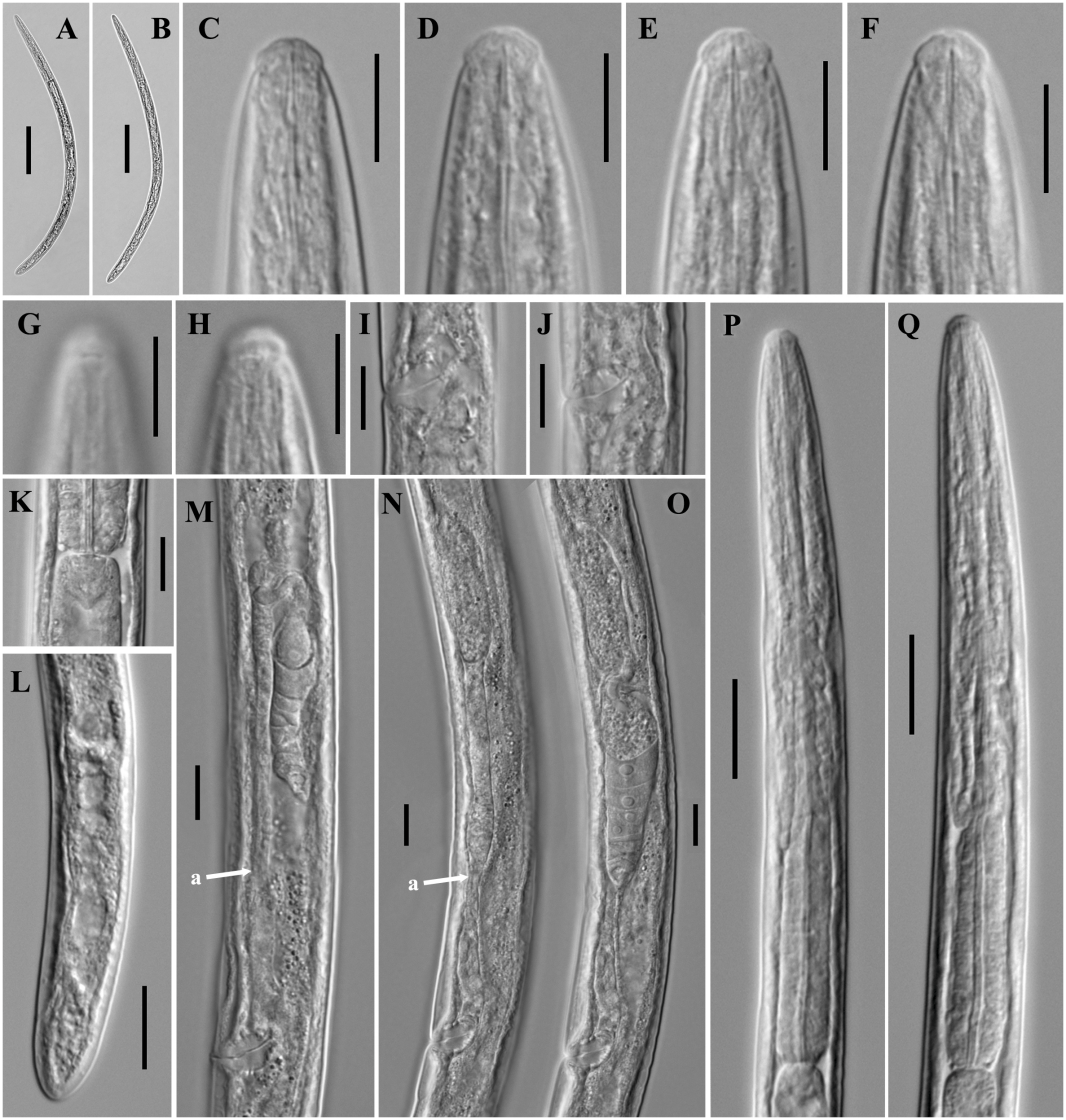
Microphotographs of *Tylencholaimus zhongshanensis* sp. nov. Female: (A, B) Entire body. (C–F) Anterior regions showing odontostyle and odontophore. (G, H) Amphidial aperture and fovea. (I, J) Vulvas in lateral view. (K) Cardia. (L) Posterior regions. (M–O) Genital branch (a: Boundary between the oviduct and uterus). (P, Q) Pharynx. Scale bars: A, B = 200 mm; L, P, Q = 20 µm; C–K, M–O = 10 µm. Holotype: G, K. Paratypes: A–F, H–J, L–Q.

**Figure 3:**
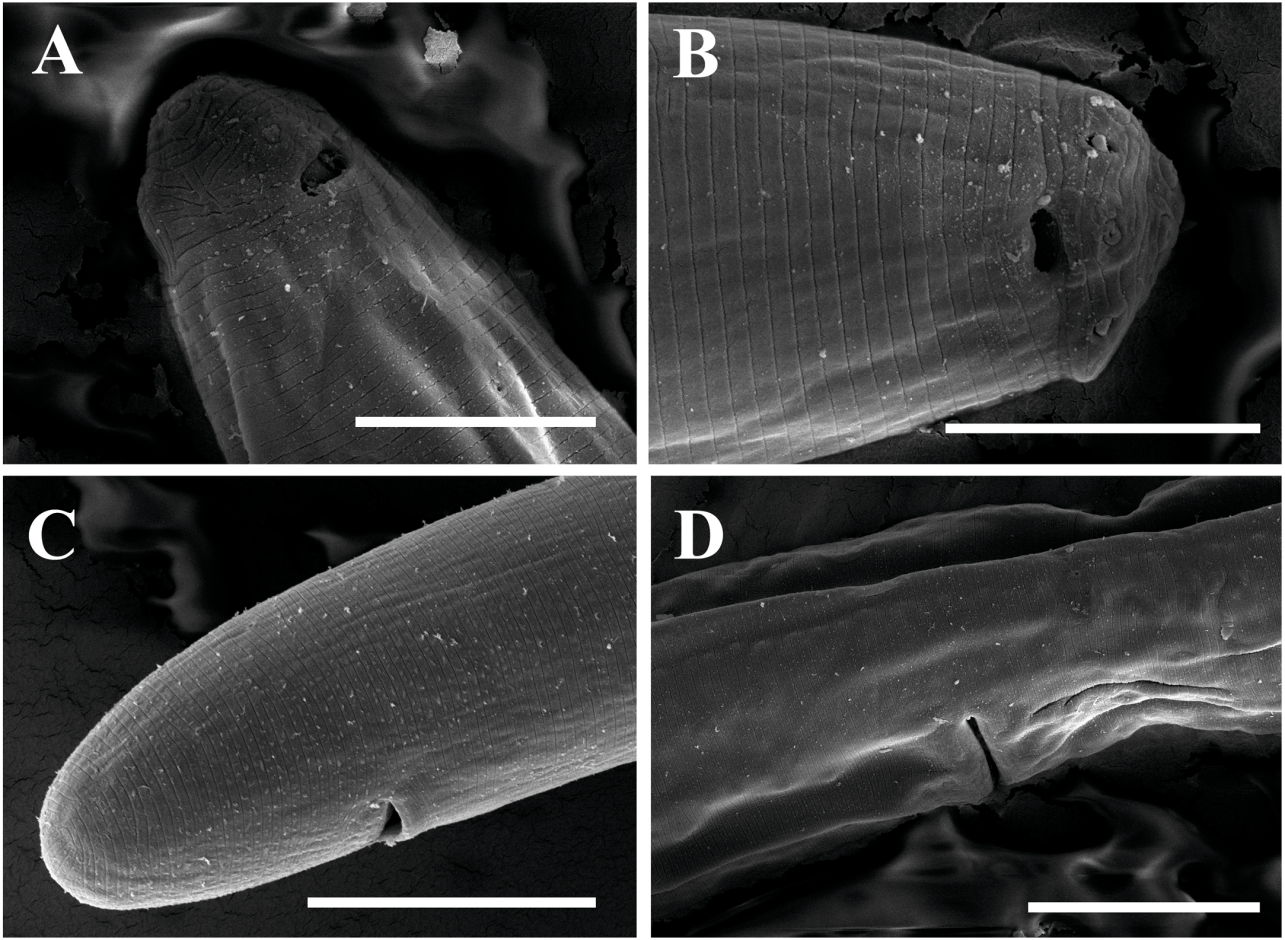
Scanning electron micrographs of * Tylencholaimus zhongshanensis* sp. nov. Female: (A, B) Lip region and amphid. (C) Posterior region. (D) Vulva. Scale bars: A, B = 5 µm; C, D = 10 µm. Paratypes: A–D.

***Tylencholaimus zhongshanensis*sp. nov.**
urn:lsid:zoobank.org:act:AFB00C8F-918E-422C-ABB6-71F4DFBCA3D5
Figs. 1–3; Table 1

### Description

Female. Measurements are listed in Table 1. Body largely cylindrical, habitus straight to ventrally curved after fixation. Cuticle two layers, 0.6–1.1 µm thick in anterior region, 1.0–1.4 µm at mid-body, and 2.1–3.0 µm on tail; outer layer with fine transverse striations, the inner one loose and shrink after fixation. Lateral chord occupying 30% in average of the body diameter at mid-body, lateral pores indistinct. Lip region cap shaped, offset from the body by a constriction, 2.3–2.8 times as wide as high or 30–38% of the body diameter at posterior end of the neck region. Lips amalgamated, labial and cephalic papillae distinct. Amphidial foveae cup shaped, opening at the level of the constriction, apertures narrow, 30% in average of the lip region width. Odontostyle slender, 0.7–0.9 times the lip region width long, its aperture one-fourth to one-third of its length. Odontophore rod-like with small basal knobs, 0.8–1.3 times as long as the odontostyle. Guiding ring single, indistinct. Nerve ring situated at 41–45% of the pharyngeal length. Anterior part of pharynx slender, basal expansion occupying 33–38% of the total pharyngeal length. Pharyngeal gland nuclei located as follows: D = 68–74%, AS1 = 41–45%, AS2 = 40–50%, PS1 = 66–80%, PS2 = 72–83%. Cardia conoid to rounded. Genital system prodelphic, postvulval sac completely absent. Ovary 30–124.5 µm long. Oviduct slender, 58–95 µm long. Junction of oviduct and uterus indistinct. Uterus simple and slender, 24–44 µm long. Sperm not observed in the genital system. Vulva transverse in ventral view. Vagina approximately 46.5–57% of the corresponding body width long, anteriorly directed. *Pars proximalis vaginae* with conoid walls, 5–8 µm long and 6–7.5 µm wide, *pars refringens* lacking, *pars distalis vaginae* 3–4 µm long. Prerectum 3.2–4.8 times and rectum 0.6–0.9 times anal body diameter long. Tail hemispheroid to elongate-hemispheroid, 1.1–1.4 times the anal body diameter long.

**Table 1:** Morphometrics of *Tylencholaimus zhongshanensis* sp. nov.

Character	Female	
	Holotype	Paratypes
n	–	9
L	600.5	550 (472.5–604.5)
a	26.8	26.4 (25.3–27.1)
b	3.9	3.6 (3.2–3.9)
c	29.0	29.6 (26.2–33.3)
c′	1.3	1.2 (1.1–1.4)
V	68.6	69.8 (68.8–71.3)
Lip region diam.	7	7 (6–7)
Lip region height	3	3 (2.6–2.9)
Amphid aperture	2	2.0 (1.9–2.3)
Odontostyle length	5	6 (5–6)
Odontophore length	7	6.0 (5–7)
Guiding ring from anterior end	4	4 ± 0.3 (4–5)
Nerve ring from anterior end	65	66 (62.5–70)
Pharyngeal length	153	152 (147–157)
Expanded part of pharynx	58	54 (49–57)
Cardia length	8	9 (6.5–11)
Body diameter at neck base	22	20 (18–23)
Body diameter at mid-body	22	21 (17–24)
Body diameter at anus	16	15 (12–17)
Anterior genital branch	133	106 (81–156)
Posterior genital branch	–	–
Vagina length	11	11.0 (10–12)
Vulva from anterior end	412	384.0 (333–417)
Prerectum length	73	62 (42–80)
Rectum length	11.5	11 (9–14)
Tail length	21	19 (16–21)

**Notes.**

All measurements are in m (except for ‘L in mm) and shown in the form: mean (minimum- maximum).

n number of specimens observed L body length a L/ maximum width b L/ pharyngeal L c L/ tail length c′ tail length/ body diameter at anus V distance of vulva from anterior end × 100/L G1 anterior uterine sac × 100/L G2 posterior genital branch × 100/L

Male. Unknown. All soil samples were processed, but no males were found.

### Habitat and locality

Rhizosphere soil of *Phalaenopsis* sp. from Zhongshan, Guangdong, China.

### Type material

Female holotype and six female paratype specimens (slide numbers: 0422627.A and 0422627.B) are deposited in the Lab of Plant Nematology/Research Center of Nematodes of Plant Quarantine, South China Agricultural University, Guangzhou, Guangdong, China, and three paratype specimens (slide numbers: 0422627.C) are deposited in the Key Laboratory of Vegetation Restoration and Management of Degraded Ecosystems, South China Botanical Garden, Chinese Academy of Sciences, Guangzhou, Guangdong, China.

### Etymology

The new species is named after its type locality, Zhongshan City.

### Diagnosis and relationships

*Tylencholaimus zhongshanensis* sp. nov. is characterized by having a body length of 473–605 µm; lip region offset and approximately one-third of the body diameter at posterior end of the neck region; amphid aperture 30% as wide as the lip region; odontostyle 5–6 µm, 0.7–0.9 times the lip region width long; odontophore rod-like with small basal knobs, 5–7 µm long, 0.8–1.3 times as long as the odontostyle; basal expansion of pharynx occupying 33–38% of the total pharyngeal length; female genital system prodelphic; postvulval sac completely absent; vulva transverse; prerectum 3.2–4.8 times anal body diameter long; tail 16–21 µm, hemispheroid to elongate-hemispheroid, 1.1–1.4 times the anal body diameter long.

All the prodelphic species of *Tylencholaimus* were compared with *Tylencholaimus zhongshanensis* sp. nov. mainly based on Vinciguerra (1986), Peña Santiago & Coomans (1994), Peña Santiago & Coomans (1996), Andrássy (2009) and Ahmad & Araki (2003). The new species is close to *T. proximus* Thorne, 1939 (Vinciguerra, 1986) with a body length approximately 0.6 mm or less, short hemispheroid tail (*c* = 24 or more), pharynx expanded behind middle, inner part of lips not offset sharply, but it can be differentiated from *T. proximus* (Peña Santiago & Coomans, 1996) mainly by having odontophore 5–7 µm (vs. 8–9.5 µm) long and 0.8–1.3 times (vs. 1.5 times) as long as the odontostyle, oviduct slender without specializations (vs. consists of a slender part and a moderately developed *pars dilatata*), posterior genital branch completely absent (vs. absent or reduced to a rudimentary sac less than one-third of the corresponding body width long) and vagina slightly directed forward (vs. transverse). The new species is close to *T. ibericus* Peña-Santiago & Coomans, 1994 (= *T. japonicus* Ahmad & Araki, 2003) in having perioral region not disc-shaped, basal part of pharynx expanded gradually, posterior genital completely absent, odontostyle less than 6 µm long and body length about 0.6 mm or less (Peña Santiago & Coomans, 1994; Andrássy, 2009), but differs by c = 26–33 (vs. 35–46, after Ahmad & Araki, 2003; 32–41, after Peña Santiago & Coomans, 1994), junction of oviduct and uterus indistinct (vs. sphincter present at the junction of oviduct and uterus, after Ahmad & Araki, 2003 and Peña Santiago & Coomans, 1994) and tail 16–21 µm (vs. 12–16 µm after Ahmad & Araki, 2003; 13–16 µm after Peña Santiago & Coomans, 1994) long without terminal caudal pore (vs. with distinct terminal caudal pore, after Ahmad & Araki, 2003).

### Molecular characterization and phylogenetic analysis

**Figure 4:**
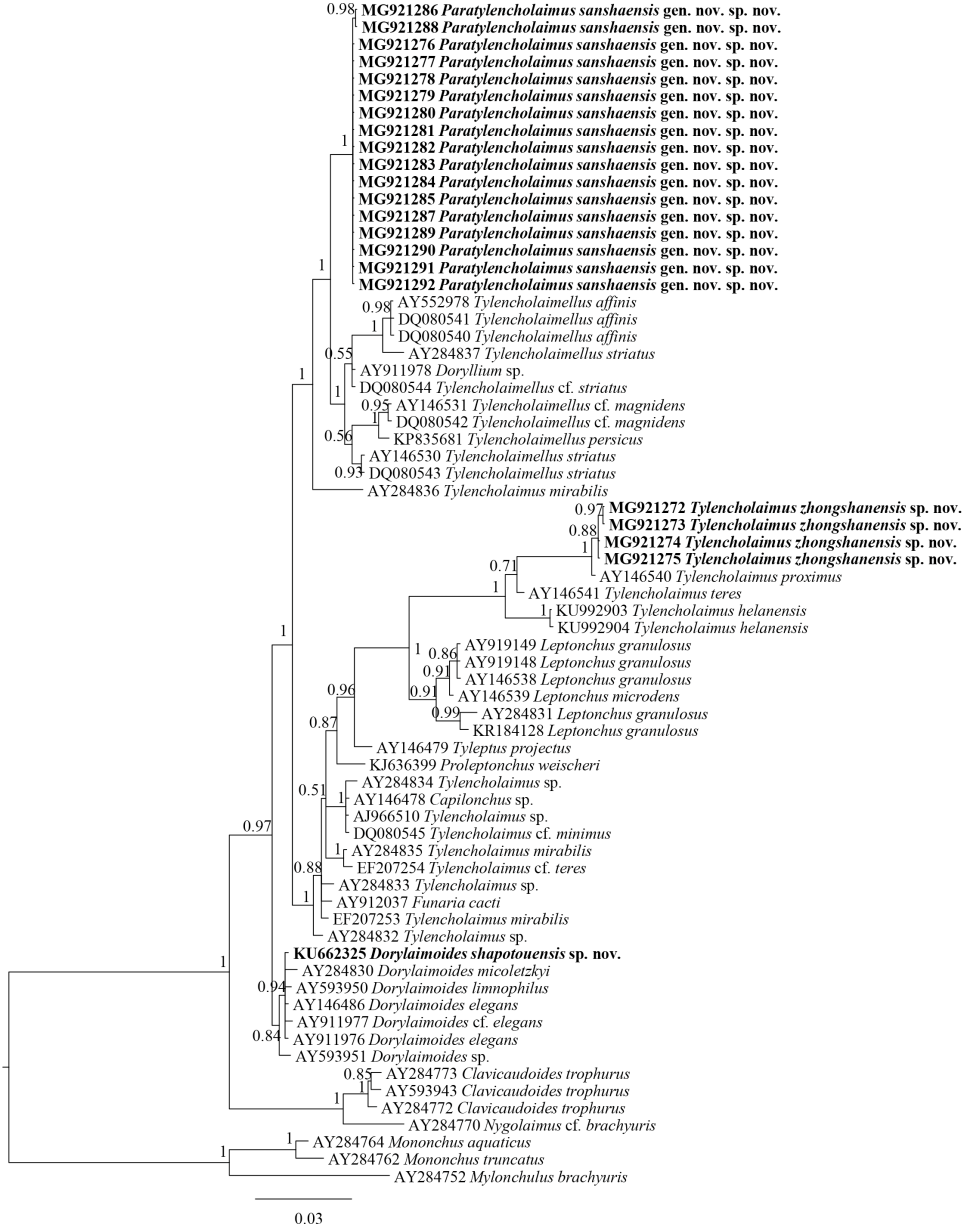
Bayesian tree of Tylencholaimellidae for 18S rDNA gene under GTR +I +G model. Posterior probabilities higher than 50% are presented for appropriate clades. Newly obtained sequences are shown in bold.

**Figure 5:**
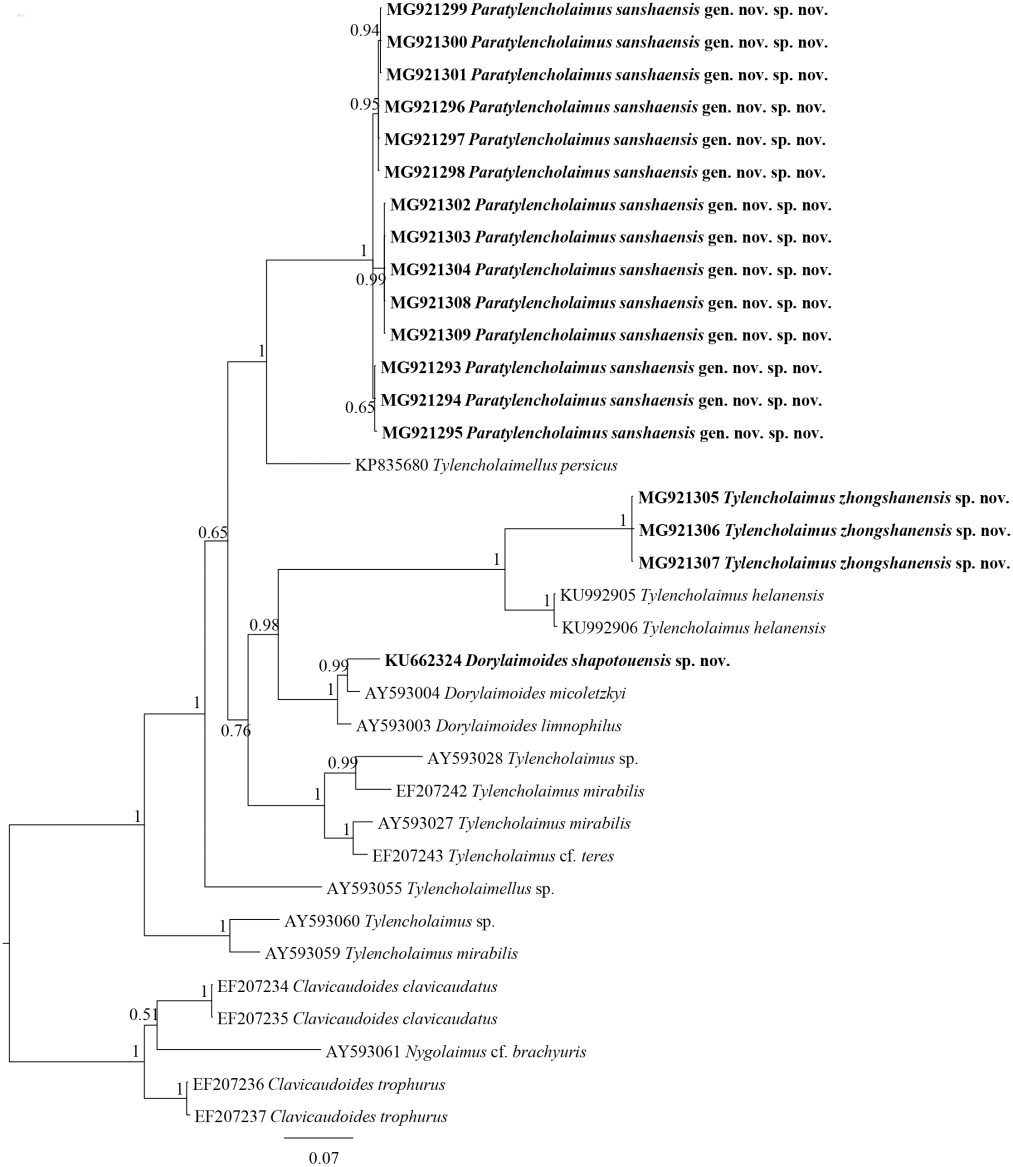
Bayesian tree of Tylencholaimellidae for D2–D3 region of 28S rDNA gene under GTR +I +G model. Posterior probabilities higher than 50% are presented for appropriate clades. Newly obtained sequences are shown in bold.

The sequences of 18S rDNA and D2–D3 region of 28S rDNA of *Tylencholaimus zhongshanensis* sp. nov. were obtained, and interindividual variabilities were both observed. Four sequences for 18S rDNA (1,747 bp; accession numbers: MG921272 to MG921275) and three sequences for the D2–D3 region of 28S rDNA (829 bp; accession numbers: MG921305 to MG921207) were deposited in GenBank. The BLAST search for the 18S rDNA showed the highest similarity (96%) to the sequences of *T. helanensis* (KU992903 and KU992904). For the D2–D3 region of 28S rDNA, both sequences showed the highest similarity (86%) to the sequences of *T. helanensis* (KU992905 and KU992906). In Bayesian trees for both the 18S rDNA and D2–D3 region of 28S rDNA (Figs. 4 and 5), the sequences of *Tylencholaimus zhongshanensis* sp. nov. formed a clade with 88% and 100% supports, respectively, and clustered together with other *Tylencholaimus* species.

In the 18S rDNA Bayesian tree (Fig. 4), *Tylencholaimus zhongshanensis* sp. nov. showed a close relationship with another prodelphic species, *T. proximus* Thorne, 1939, with 100% support. These two species are also close to each other in morphology but can be separated mainly by the characteristics of odontostyle, odontophore and genital system structures as discussed above. In the 28S rDNA Bayesian tree (Fig. 5), *Tylencholaimus zhongshanensis* sp. nov. showed a close relationship with *T. helanensis*. However, *Tylencholaimus zhongshanensis* sp. nov. can be differentiated from *T. helanensis* mainly by body length and shorter odontostyle (473–605 µm vs. 0.93–1.07 mm; 5–6 µm vs. 8–9.5 µm), and female prodelphic (vs. didelphic-amphidelphic) (Wu et al., 2018) although the sequences showed the highest similarity to each other.

**Table:** 

**Figure 6:**
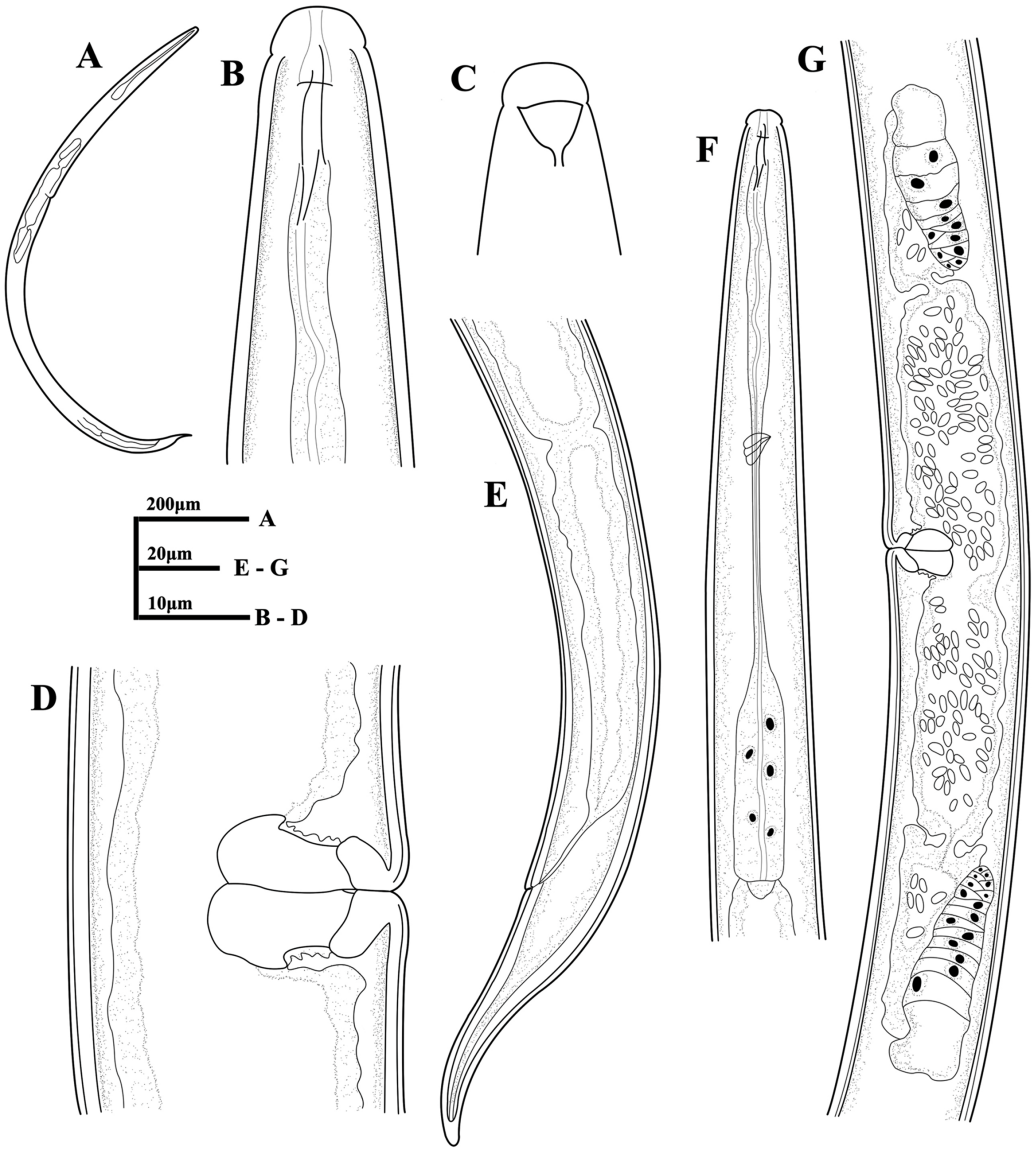
Ink drawing of *Dorylaimoides shapotouensis* sp. nov. Female: (A) Entire body. (B) Anterior region. (C) Amphidial fovea. (D) Vulva in lateral view. (E) Posterior region. (F) Pharynx. (G) Genital system. Holotype: E. Paratypes: A–D, F, G.

**Figure 7:**
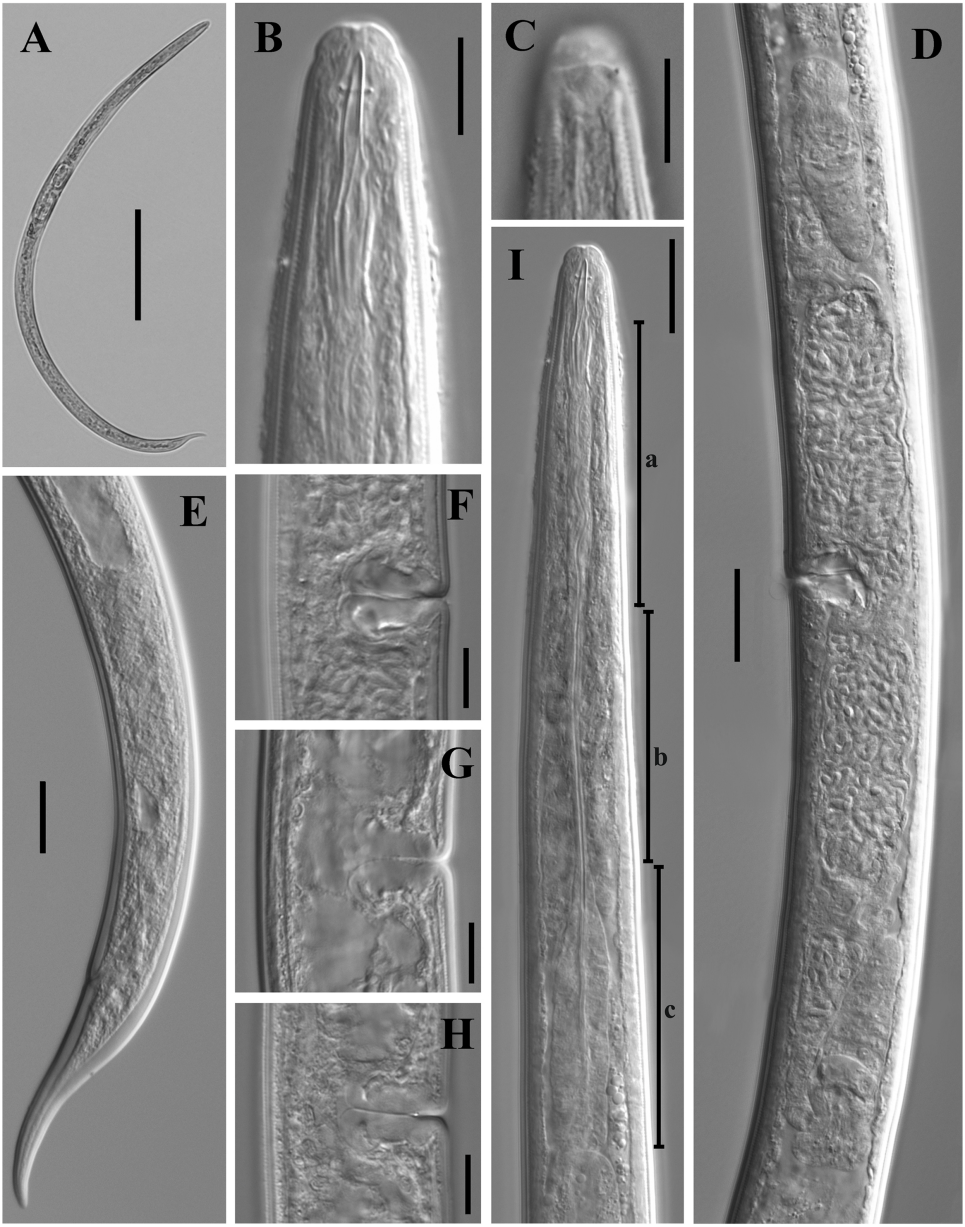
Microphotographs of *Dorylaimoides shapotouensis* sp. nov. Female: (A) Entire body. (B) Anterior region. (C) Amphidial fovea. (D) Genital system. (E) Posterior region. (F–H) Vulvas in lateral view. (I) Pharynx (a, slender anterior part; b, isthmus-like portion; c, basal expansion). Scale bars: A = 200 µm; B, C, F–H = 10 µm; D, E, I = 20 µm. Holotype: E. Paratypes: A–D, F–I.

**Figure 8:**
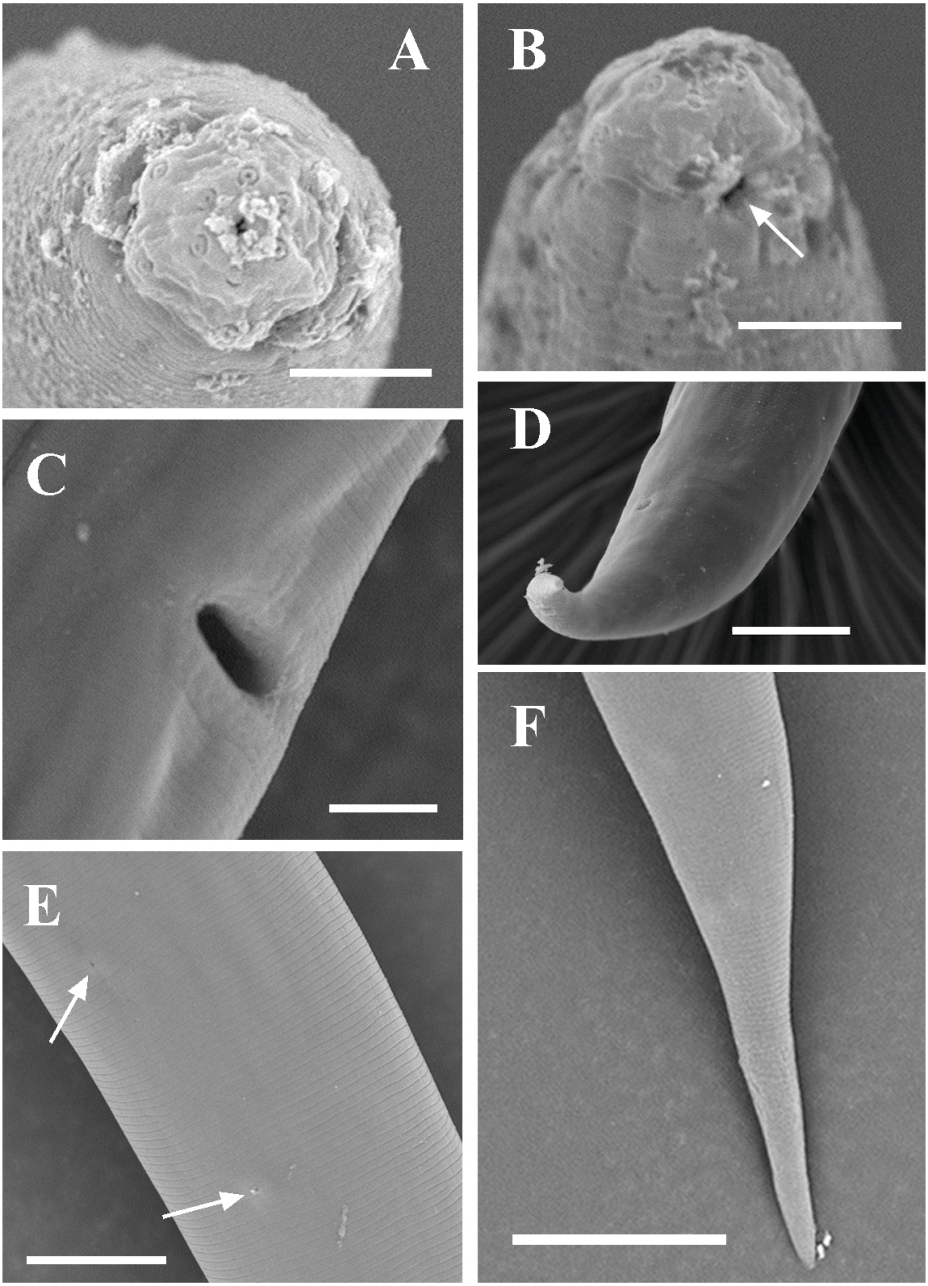
Scanning electron micrographs of *Dorylaimoides shapotouensis* sp. nov. Female: (A) Lip region. (B) Anterior end showing amphidial aperture (arrowed). (C) Vulva. (D, F) Posterior region. (E) Cuticle and body pores (arrowed). Scale bars: A–C = 4 µm; D, E = 10 µm; F = 20 µm. Paratypes: A–F.

***Dorylaimoides shapotouensis*sp. nov.**
urn:lsid:zoobank.org:act:F6CB7E8A-0C6D-496F-9B98-2E151328E64F
Figs. 6– 8; Table 2

### Description

Female. Measurements are listed in Table 2. Body slender, ventrally curved showing an open ‘C’ shaped after fixation. Cuticle with fine transverse striations, 0.6–1.6 µm thick in anterior region, 1.2–2.1 µm at mid-body, and 3.2–3.9 µm on tail. Lateral chord occupying 14–17% of the body diameter at mid-body. Lip region rounded, offset by a constriction, about 2.5 times as wide as high or about 0.3 times as wide as body diameter at posterior end of pharyngeal region. Lips practically amalgamated, labial papillae protruding and can be seen easily in SEM. Amphidial foveae cup shaped, opening at the level of the constriction, apertures about 0.6 times as wide as lip region width. Odontostyle asymmetrical, with a distinct lumen. Odontophore arcuated, narrowing posteriorly, about 0.8 times as long as the odontostyle. Guiding ring distinct and single. Nerve ring situated at 40–51% of the pharyngeal length. Pharynx three parts, including an anterior part slender, a much narrower isthmus-like portion and a cylindrical basal expansion, basal expansion occupying 21–28% of the total pharyngeal length. Pharyngeal gland nuclei located as follows: D = 69–82%, AS1 = 26–42%, AS2 = 34–49%, PS1 = 50–66%, PS2 = 58–74%. Cardia short, rounded. Genital system didelphic-amphidelphic. Ovary reflexed, usually reaching the junction of oviduct and uterus, anterior one 42.5–46 µm long and posterior one 31–59.5 µm long. Oviduct consists of a wider *pars dilatata* and a slender part, anterior one 32–63 µm long and posterior one 46–53 µm long. Sphincter present at the junction of oviduct and uterus. Uterus simple and with a wide lumen, anterior 53–65 µm long and posterior 45–75 µm long. A lot of sperm in the uterus and a few cells in the *pars dilatate* of oviduct serving as spermatheca. Vulva transverse. Vagina extending 45–56.5% inwards the corresponding body width. *Pars proximalis vaginae* with thick walls, 12–14 µm long and 11–13 µm wide, *pars refringens* lacking. Prerectum 3.3–5.9 times and rectum 0.9–1.5 times the body diameter at anus long. Tail elongate conoid with rounded terminus, posterior region bent dorsally, 2.8–3.9 times the anal body diameter long.

Male. Unknown. All soil samples were processed, but no males were found.

### Habitat and locality

Rhizosphere soil of apple trees from Shapotou Region, Zhongwei City, the Ningxia Hui Autonomous Region, China; GPS coordinate 104°59.723′E, 37°28.153′N.

**Table 2:** Measurements of *Dorylaimoides shapotouensis* sp. nov. and eight populations of *D. micoletzkyi*.

Character	*Dorylaimoides shapotouensis* sp. nov.		*D. micoletzkyi*	
	Holotype	Paratypes 6♀♀	Peralta & Peña Santiago (1995)5♀♀	Other seven populations (Peña Santiago & Peralta, 1997b) more than 34 ♀♀ + 11♂♂
L	1.10	1.08 (1.02–1.13)	1.20 (1.10–1.26)	0.9–1.7
a	33.7	34.9 (32.6–36.9)	40.3 (36.7–44.7)	30–44
b	5.3	5.6 (5.3–5.8)	6.50 (6.10–7.10)	
c	16.0	16.9 (14.8–19.9)	18.5 (16.4–19.4)	12.5–29
c’	3.6	3.3 (2.8–3.9)	3.60 (3.4–4.2)	2.2–4.5
V	38.0	40.3 (38.5–42.5)	40.3 (38.8–41.8)	39–44
Lip region diam.	8	8 (8–9)	8.5–9.5	8–10
Lip region height	4	3 (3–4)	3.5–4.0	
Amphid aperture	4.5	5.5 (5.0–6)	7.0	
Odontostyle length	11	11 (9–13)	7.10 (7.0–7.5)	6–10
Odontophore length	8	8.5 (8–9.5)	18.2 (17.5–19.0)	16–17
Guiding ring from anterior end	7	7 (6–7)	7.0–7.5	
Nerve ring from anterior end	94	93 (88–99)	89.4 (87.0–94.0)	
Pharyngeal length	207	193 (178–209)	183 (177–189)	
Expanded part of pharynx	45	49 ± 4.0 (43–54)	53.7 (50–56)	50–65
Cardia length	8	8 (5–11)	6–8	
Body diameter at neck base	28	27 (25–31)	26.7 (25.0–27.5)	
Body diameter at mid-body	33	31.0 (29–34)	29.8 (28.0–32.5)	
Body diameter at anus	19	20 (19–22.0)	18.2 (17.0–19.5)	
Anterior genital branch	129	109 (89–133)	150 (134–169)	
Posterior genital branch	135	108.0 (81–126)	160 (138–178)	
Vagina length	17	17 (15–18)	13–15	
Vulva from anterior end	419	436 (413–451)	483 (460–500)	
Prerectum length	85	99 (62–118)	97.0 (78–112)	
Rectum length	25	22 (19–28)	22.0 (20.0–25.0)	
Tail length	69	65 (53–73)	65.0 (59.0–75.0)	47–84 (female)

**Notes.**

All measurements are in m (except for ‘L in mm) and shown in the form: mean (minimum- maximum).

n number of specimens observed L body length a L/ maximum width b L/ pharyngeal L c L/ tail length c′ tail length/ body diameter at anus V distance of vulvafrom anterior end × 100/L G1 anterior uterine sac × 100/L G2 posterior genital branch × 100/L

### Type material

Female holotype and four female paratype specimens (slide numbers: M72.A and M72.B) are deposited in the Lab of Plant Nematology/Research Center of Nematodes of Plant Quarantine, South China Agricultural University, Guangzhou, Guangdong 510642, China, and two female paratype specimens (slide numbers: M72. C) are deposited in the Key Laboratory of Vegetation Restoration and Management of Degraded Ecosystems, South China Botanical Garden, Chinese Academy of Sciences, Guangzhou, Guangdong 510642, China.

### Etymology

The new species is named after Shapotou Region, a successful soil restoration area in China.

### Diagnosis and relationships

*Dorylaimoides shapotouensis* sp. nov. is characterized by having a body length of 1.02–1.13 mm; lip region rounded and offset by a constriction, lips practically amalgamated, labial papillae protruding; odontostyle 9–13 µm, asymmetrical with a distinct lumen, odontophore arcuated and narrowing posteriorly, 8–9.5 µm; basal expanded part of pharynx occupying 21–28% of the total pharyngeal length; genital system didelphic-amphidelphic; prerectum 3.3–5.9 times and rectum 0.9–1.5 times the body diameter at anus long; tail elongate conoid with rounded terminus and the posterior region bent dorsally, 53–73 µm, 2.8–3.9 times the anal body diameter long.

The new species is close to *D. leptus* Husain & Khan, 1968, *D. siddiqii* Baqri & Khera, 1979, *D. micoletzkyi* (de Man, 1921) Thorne & Swanger, 1936 and *D. punctatus* Khan & Park, 1999 in having a didelphic genital system and a conoid elongate tail dorsally bent at the end and longer than 45 µm based on the key provided by Pedram, Pourjam & Vinciguerra (2011). It differs from *D. leptus* (Husain & Khan, 1968) by odontostyle longer (9–13 µm vs. 8–9 µm), odontophore shorter (8–9.5 µm vs. 11–12 µm), vulva located more anterior (V= 38–42.5 vs. 45–48), prerectum longer (62–118 µm vs. 35 µm). It differs from *D. siddiqii* (Baqri & Khera, 1979) with longer odontostyle (9–13 µm vs. 8–9 µm), shorter odontophore (8–9.5 µm vs. 11–13µm), larger c’ value (c’ = 2.8–3.9 vs. 2.5–2.8), and males unknown (vs. present). From *D. micoletzkyi* (Peralta & Peña Santiago, 1995), the new species differs by amphid opening narrower (about 0.6 times vs. 0.7–0.8 times as wide as lip region width), odontophore shorter (8–9.5 µm vs. 16–17 µm, after (Jana & Baqri, 1981); vs. 17.5–19.0 µm; and about 0.8 vs. 2.3–2.7 times the odontostyle long, after (Peralta & Peña Santiago, 1995), pharynx consists of an anterior part slender, a much narrower isthmus-like portion, a cylindrical basal expansion (vs. pharynx consists of a slender anterior part and a cylindrical basal bulb), genital system shorter (anterior branch 89–133 µm vs. 134–169 µm; posterior branch 81–135 µm vs. 138–178 µm) and males unknown (vs. present). The new species differs from *D. punctatus* (Khan & Park, 1999) by having a shorter body length (*L* = 1.02–1.13 mm vs. 1.3–1.4 mm), lower ‘a’ value (*a* = 32.6–36.9 vs. 39.5–45.7), odontostyle longer (9–13 µm vs. 7.3–8.0 µm), tail shorter (53–73 µm vs. 77–83 µm) and cuticle with fine transverse striations (vs. cuticle marked with zig-zag lines throughout the body).

### Molecular characterization and phylogenetic analysis

Each sequence of 18S rDNA and D2–D3 region of 28S rDNA of *Dorylaimoides shapotouensis* sp. nov. (1,743 bp and 825 bp, respectively) was obtained and deposited in GenBank (accession numbers: KU662325 for the 18S rDNA and KU662324 for the D2–D3 region of 28S rDNA). The BLAST search for the 18S rDNA showed the highest similarity (99%) to the sequence of *D. micoletzkyi* (AY284830) and showed 8 nucleotide differences. The D2–D3 region of 28S rDNA showed the highest similarity (95%) to the sequences of *D. micoletzkyi* (AY593004) with 40 nucleotide and 4 gaps differences. In the 18S rDNA phylogenetic reconstructions (Fig. 4), the new species clustered together with other species of *Dorylaimoides* with 84% support. In the D2–D3 region of 28S rDNA phylogenetic reconstructions (Fig. 5), the new species is located in a 100% supported clade with *D. micoletzkyi* and *D. limnophilus* (an opisthodelphic species).

Most measurements of *Dorylaimoides shapotouensis* sp. nov. overlap those of eight documentary populations of *D. micoletzkyi* (Table 2), but *Dorylaimoides shapotouensis* sp. nov. can be easily differentiated from *D. micoletzkyi* mainly by the pharynx morphology and the odontophore length. Peña Santiago & Peralta (1997a) and Peña Santiago & Peralta (1997b) published a series of papers on the genus *Dorylaimoides*, and comprehensively discussed the suitability of using the female genital system types and the tail to identify the species of *Dorylaimoides* and made a key to the species and groups based on the female genital system types (didelphic, opisthodelphic and pseudodidelphic). In the 28S rDNA Bayesian tree, *Dorylaimoides shapotouensis* sp. nov. showed a closer relationship with another didelphic species, *D. micoletzkyi* rather than with the opisthodelphic species *D. limnophilus*. However, the inner relationships of *Dorylaimoides* remain unclear in the 18S rDNA Bayesian tree. Thus, to clarify the evolutionary relationships among the three groups with different genital system types, more molecular data of *Dorylaimoides* are needed.

**Table:** 

***Paratylencholaimus*** **gen. nov.**
urn:lsid:zoobank.org:act:4BFCC48B-38E2-449C-B1F5-338E46E7B099

### Diagnosis

Tylencholaimellidae. Paratylencholaiminae subfam. nov. Cuticle dorylaimoid with fine transverse striations. Lip region cap-shape, offset from the body. Amphidial fovea not sclerotized. Odontostyle straight, tubular with small aperture, without dorsal accessory pieces. Odontophore rod-like and basal part slightly expanded. Guiding ring simple. Pharynx slender in anterior part, the basal part expanded occupying one-third of the total pharyngeal length. Female genital system didelphic. Vulval lips not sclerotized. Tail short, rounded to conoid-round. Males unknown.

### Relationships

*Paratylencholaimus* gen. nov. is close to *Goferus* (Jairajpuri & Ahmad, 1992) and *Phellonema* Thorne, 1964 in having simple amphidial fovea, odontostyle without stiffening pieces, female didelphic (Andrássy, 2009). From *Goferus* (Jairajpuri & Ahmad, 1992; Andrássy, 2009), the new genus can be differentiated by having lip region offset (vs. practically continuous), odontophore rod-like (vs. arcuate), posterior third of pharynx with a cylindrical basal expansion (vs. much short and pyriform) and tail rounded to conoid-round (vs. elongate-rounded). From *Phellonema* (Jairajpuri & Ahmad, 1992; Andrássy, 2009), the new genus differs by having lip region offset from body (vs. continuous), basal part of odontophore slightly expanded (vs. with strongly developed flanges), basal part of pharynx expanded gradually (vs. constricted) and anus not subterminal (vs. anus subterminal).

### Etymology

The new genus is named as *Paratylencholaimus* (latin *para*- = similar), as its inner characteristics are similar to those of the genus *Tylencholaimus*, but it has a different type of cuticle which is dorylaimoid.

### Type and only species

**Table:** 

**Figure 9:**
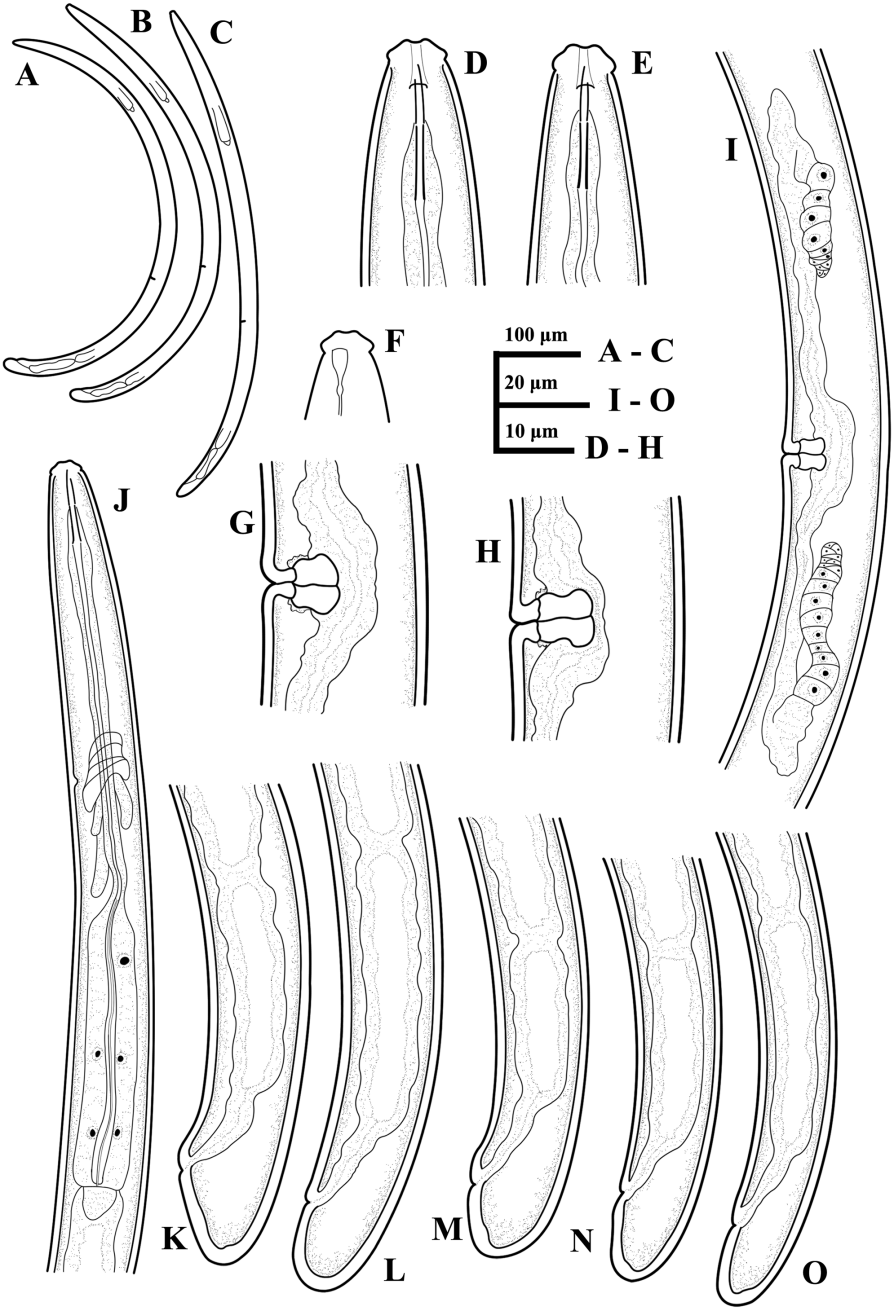
Ink drawing of *Paratylencholaimus shanshaensis* gen. nov. sp. nov. Female: (A–C) Entire bodies. (D, E) Anterior regions. (F) Amphid. (G, H) Vulvas in lateral view. (I) Genital system. (J) Pharynx. (K–O) Posterior regions. Holotype: E, L. Paratypes: A–D, F–K, M–O.

**Figure 10:**
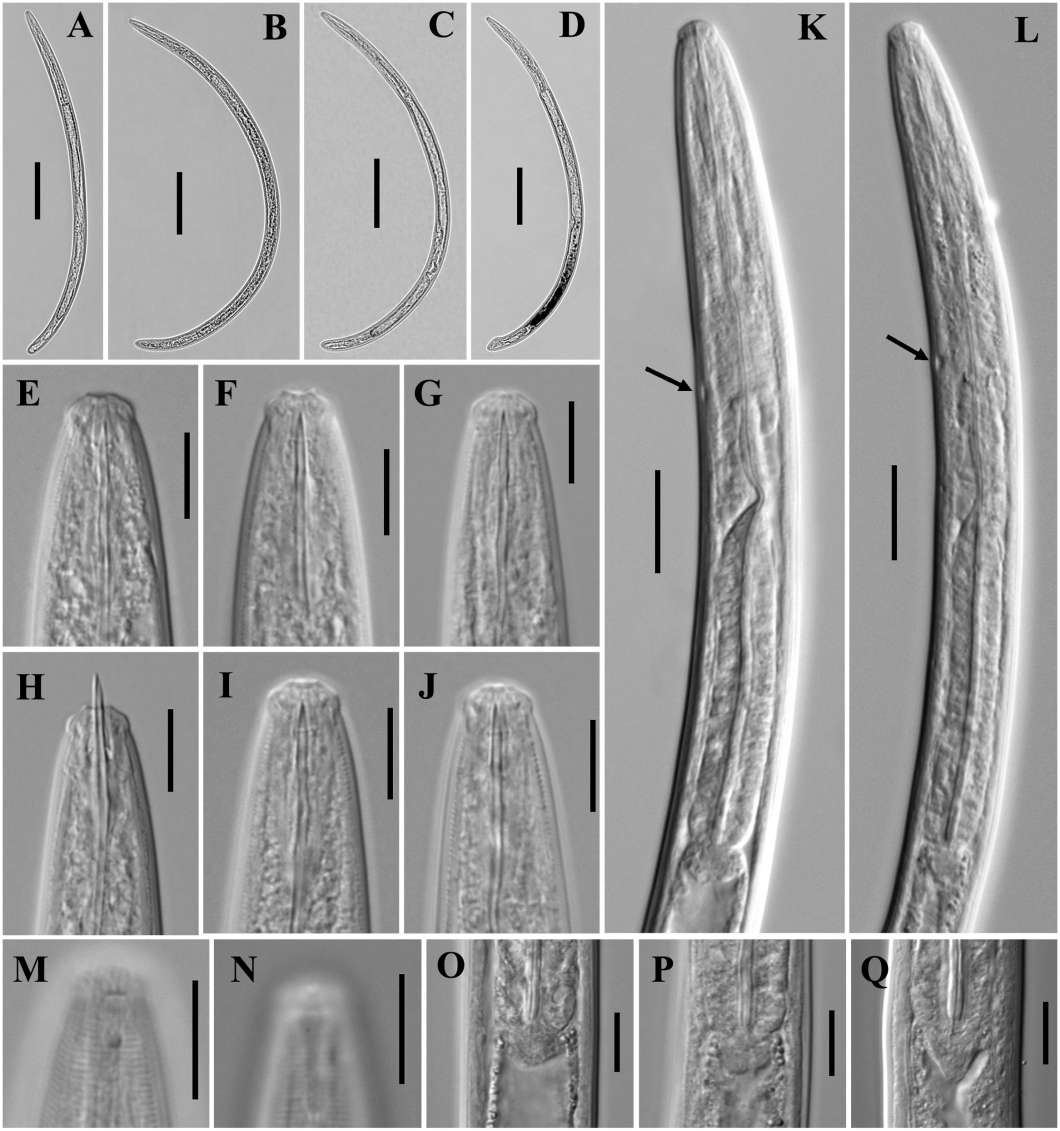
Microphotographs of *Paratylencholaimus shanshaensis* gen. nov. sp. nov. Female: (A–D) Entire bodies. (E–J) Anterior regions. (K, L) Pharynx (arrowed: hemizonid). (M, N) Amphids. (O–Q) Cardias. Scale bars: A–D = 100 µm; K, L = 20 µm; E–J, M–Q = 10 µm. Paratypes: A–Q.

**Figure 11:**
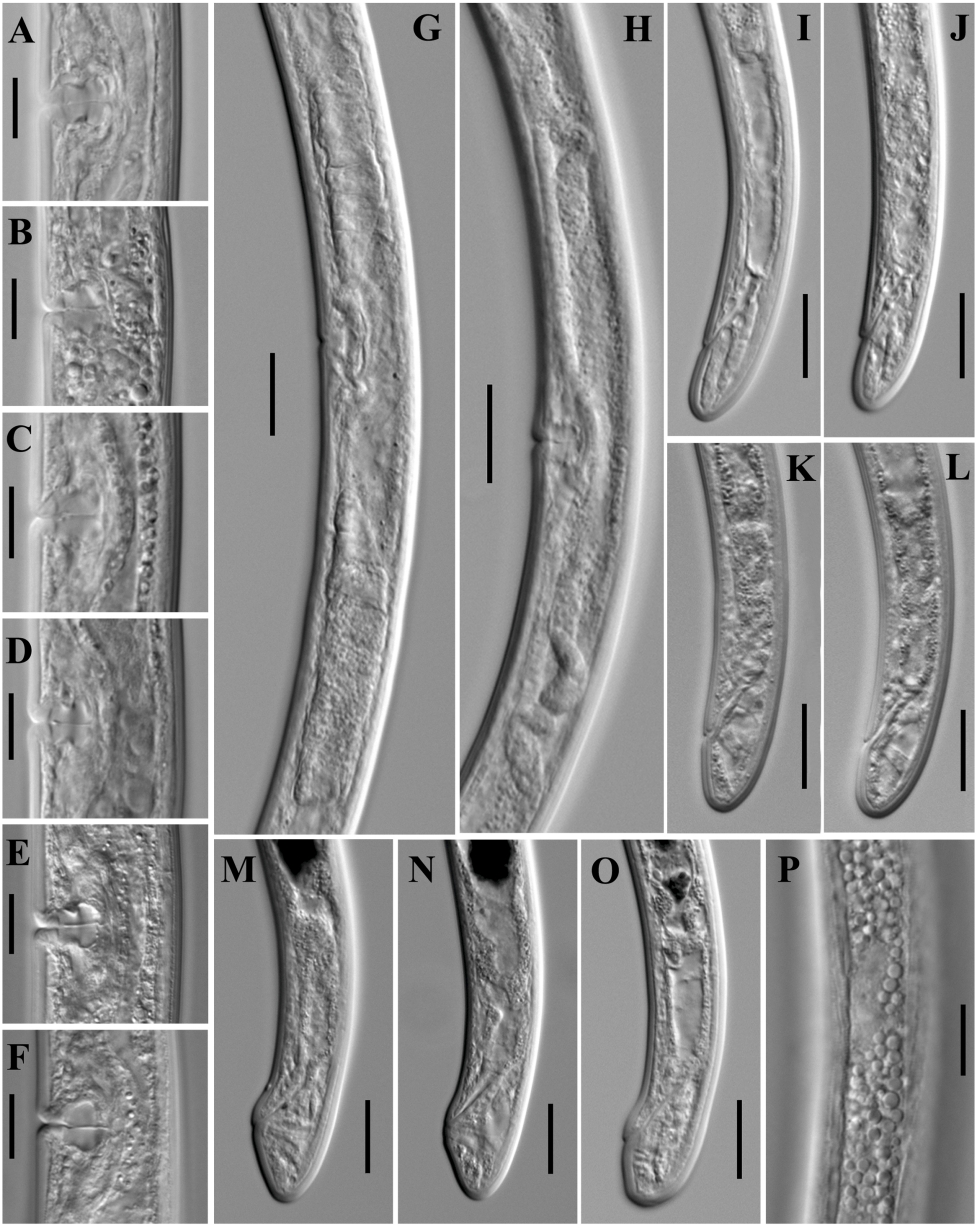
Microphotographs of *Paratylencholaimus shanshaensis* gen. nov. sp. nov. Female: (A–F) Vulvas in lanteral view. (G, H) Genital systems. (I–O) Posterior regions. (P) Lateral chord at mid-body. Scale bars: G, H = 20 µm; A–F, I–P = 1 µm. Paratypes: A–P.

**Figure 12:**
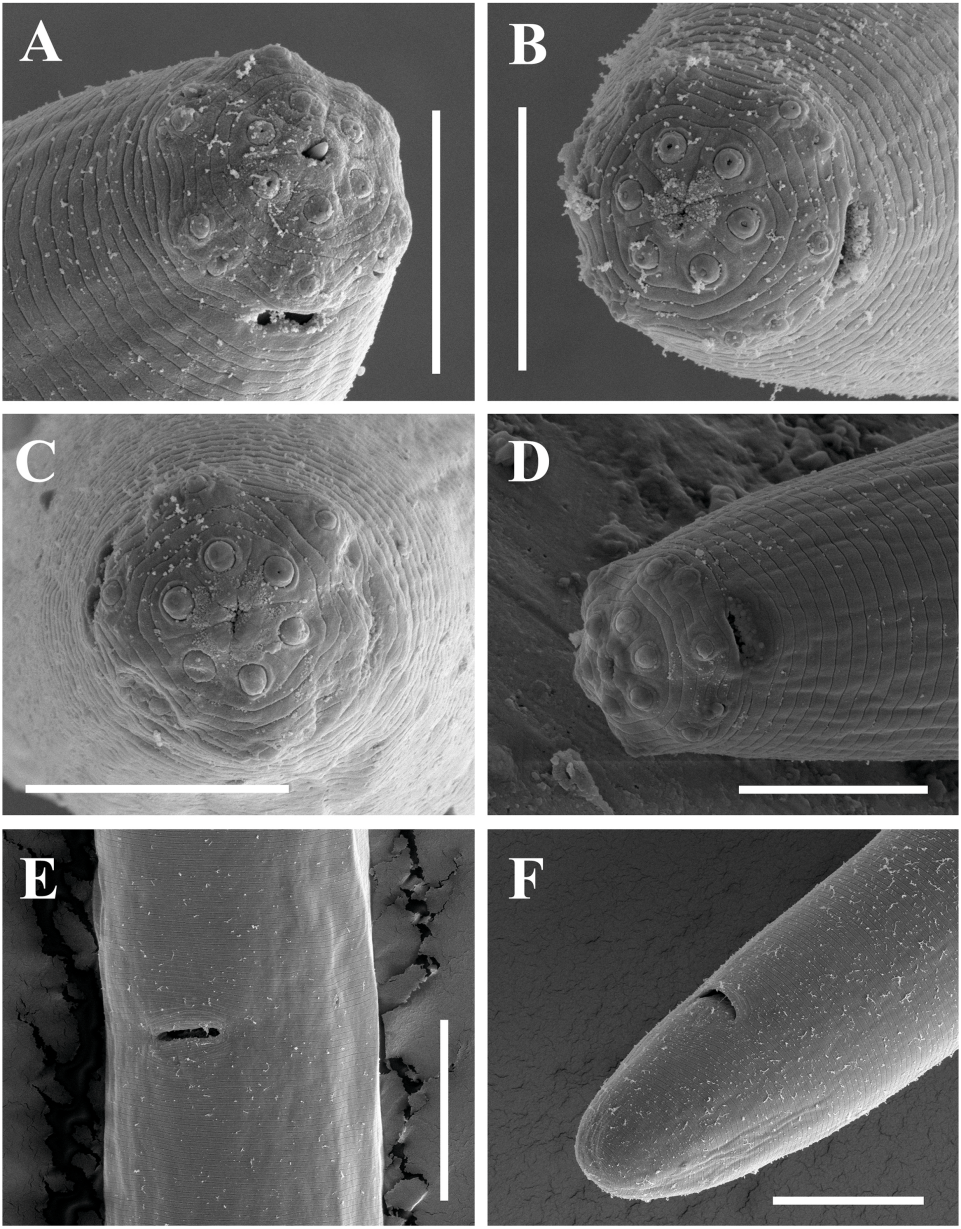
Scanning electron micrographs of *Paratylencholaimus shanshaensis* gen. nov. sp. nov. Female: (A–C) Lip regions. (D) Amphid. (E) Vulva. (F) Posterior region. Scale bars: A–D = 5 µm; E, F = 10 µm. Paratypes: A–F.

***Paratylencholaimus sanshaensis*** **gen. nov. sp. nov.**
urn:lsid:zoobank.org:act:63A581ED-4755-481F-B7A9-F71C79946A9C
Figs. 9–12; Table 3

### Descriptions

Female. Measurements are listed in Table 3. Body cylindrical, habitus curved ventrally on different levels into an open ‘C’ shape after fixation. Cuticle with fine transverse striations, 0.8–1.4 µm thick in anterior region, 0.9–2.1 µm at mid-body and 1.9–3.1 µm on tail. Lateral chord occupying about one-third of the body diameter at mid-body, lateral pores indistinct. Lip region cap shaped, offset from the body, 2.1–3.5 times as wide as high or about one-third of the body diameter at posterior end of the neck region. Lips largely amalgamated, inner part of lips separated, labial and cephalic papillae distinct and labial papillae larger than the cephalic ones. Amphideal fovea goblet-shaped, its apertures quite small, about one-fourth as wide as the lip region. Odontostyle straight with distinct lumen and aperture, 0.8–1.2 times as long as the lip region width, its aperture about one-third of its length. Odontophore rod-like, 0.8–1.4 times as long as the odontostyle. Guiding ring single. Nerve ring situated at 37.5–51% of the pharyngeal length. Hemizonid occurs at the level of nerve ring. Anterior part of pharynx slender, basal part occupying 34–45% of the total pharyngeal length, expand gradually and its anterior end tend to tilt dorsally. Pharyngeal gland nuclei located as follows: D = 64–72.5%, AS1 = 22–45%, AS2 = 31–56%, PS1 = 55–79%, PS2 = 64–86%. Cardia conoid to elongate-conoid. Genital system didelphic-amphidelphic. Ovary reflexed, anterior one 19–64.5 µm and posterior one 26–79 µm long. Oviduct slender, anterior 35–83 µm and posterior one 23.5–67 µm long. Junction of oviduct and uterus indistinct. No sperm present. Uterus simple and slender, anterior 15–49 µm and the posterior one 17–55 µm long. Vulva transverse. Vagina extending 33.5–56% inwards the corresponding body width. *Pars proximalis vaginae* 5–8 µm long and 5–9 µm wide, *pars refringens* lacking, *pars distalis vaginae* 2–4 µm long. Prerectum 1.2–4.4 times and rectum 0.8–1.7 times anal body diameter long. Anal region ventrally flattened to distinctly bulge. Tail rounded to conoid-round, 0.8–1.4 times anal body diameter long.

Male. Unknown. All soil samples were processed, but no males were found.

### Etymology

The new species is named after the Sansha City, which is its type locality.

### Type material

Female holotype, twelve female paratype specimens (slide numbers: B1a.A, B1a.B, B1a.C, B1a.D and B1a.E) and 44 female from Huadu and Zhongshan (slide numbers: HuaDu.61.A–C, 0422627.D–I and 0624601.A–E) are deposited in the Lab of Plant Nematology/Research Center of Nematodes of Plant Quarantine, South China Agricultural University, Guangzhou, Guangdong 510642, China, and five female from Boluo (slide numbers: BoLuo.A and Boluo.B) are deposited in the Key Laboratory of Vegetation Restoration and Management of Degraded Ecosystems, South China Botanical Garden, Chinese Academy of Sciences, Guangzhou, Guangdong 510642, China.

### Type habitat and locality

Rhizosphere soil of *Euphorbia* sp. from Yongxing Island, Sansha City, Hainan, China.

### Other habitat and localities

Culture medium of *Scindapsus* sp. from Huadu District, Guangzhou, Guangdong, China; culture medium of *Phalaenopsis* sp. from Zhongshan, Guangdong, China; rhizosphere soil of *Citrus* sp. from Boluo County, Huizhou, Guangdong, China.

### Diagnosis

*Paratylencholaimus sanshaensis* gen. nov. sp. nov. is characterized by having body 581–772 mm long; lip region cap-shaped and offset from the body; amphid apertures quite small; odontostyle straight, 7–11 µm long and 0.8–1.2 times the lip region width long; odontophore rod-like, 6–11 µm, 0.8–1.4 times as long as the odontostyle; hemizonid occurs at the level of nerve ring; pharyngeal basal bulb occupying 34–45% of the total pharyngeal length; oviduct and uterus slender without differentiations, junction of oviduct and uterus indistinct; vulva transverse; *pars refringens* lacking; prerectum 1.2–4.4 times and rectum 0.8–1.7 times anal body diameter long; tail rounded to conoid-round, 15–21 µm long, 0.8–1.4 times the anal body diameter long.

### Feeding type

One noticed phenomenon was observed in the Zhongshan population of *Paratylencholaimus sanshaensis* gen. nov. sp. nov. The anus of this population showed morphological diversity: nematodes with normal color intestines have a flat anal region, while the nematodes with a black mass in intestines have bulge anus. The culture medium of *Phalaenopsis* sp., which this population is collected from, consists of three levels: sphagnum on the top, coco coir in the middle and barks at the bottom. It can be suggested that the nematodes with a black mass in intestines may feed on the barks. This finding indicates that the food source can influence the morphology of nematodes, and the feeding type of *Paratylencholaimus sanshaensis* gen. nov. sp. nov. is omnivorous.

### Molecular characterization and phylogenetic analysis

The sequences of 18S rDNA and the D2–D3 region of 28S rDNA of *Paratylencholaimus sanshaensis* gen. nov. sp. nov. were obtained. The interindividual variabilities were observed both in the 18S rDNA and D2–D3 region of 28S rDNA. Seventeen sequences for 18S rDNA **(**1,743 bp**)** and fourteen sequences for 28S rDNA **(**827 bp) were deposited in GenBank (accession numbers: MG921276 to MG921292 for 18S rDNA, MG921293 to MG921309 for 28S rDNA). 1 bp interindividual variability was observed only in 18S rDNA. The BLAST search for the 18S rDNA sequences showed the highest similarity (98%) to the sequence of an unidentified species (EF024986). The D2–D3 region of 28S rDNA showed the highest similarity (85%) to the sequences of *Dorylaimus* sp. (KP954677). In both the 18S rDNA and D2–D3 region of 28S rDNA Bayesian trees (Figs. 4 and 5), the sequences of *Paratylencholaimus sanshaensis* gen. nov. sp. nov. clustered together and formed a clade with 100% support, and showed a close relationship with the species of Tylencholaimellidae.

**Table 3:** Measurements of *Paratylencholaimus sanshaensis* gen. nov. sp. nov.

Character	Type material (Sansha population)		Huadu population	Zhongshan population	Boluo population	Total range
	Holotype	Paratypes 12 ♀♀	10 ♀♀	34 ♀♀	5 ♀♀	62 ♀♀
L	650	690 (636–772)	678 (595.0–757)	662 ± 39.5 (581–745)	681.0 (633–746)	581–772
a	28.4	28.9 (27.6–30.7)	30.8 (27.6–32.6)	30.3 ± 2.1 (26.3–35.5)	30.1 (25.3–33.2)	25.3–35.5
b	3.4	3.8 (3.2–4.4)	3.9 (3.3–5.2)	3.9 ± 0.2 (3.6–4.2)	3.8 (3.6–3.9)	3.2–5.2
c	36.5	37.7 (33.4–42.3)	37.7 (34.8–41.6)	38.0 ± 2.4 (33.1–43.3)	38.5 (34.9–43.7)	33.1–43.7
c′	1.1	1.1 (0.9–1.4)	1.1 (0.9–1.2)	1.0 ± 0.1 (0.8–1.2)	1.1 (0.9–1.2)	0.8–1.4
V	63.9	61.0 (59.1–63.1)	61.3 (60.0–63.1)	60.2 ± 1.0 (58.3–62.2)	61.2 (59.4–62.4)	58.3–63.9
Lip region diameter	9	8 (8–9)	8 (7–8.5)	8 ± 0.3 (7.0–8)	8 (7–8)	7–9
Lip region height	2.5	3 (2–3)	3.0 (2.5–3)	3 ± 0.2 (2–3.5)	3 (3–3.5)	2–3.5
Amphid aperture	2	2 (2–3)	2.0 (2–4)	2 ± 0.2 (2–2.5)	2.0 (1.5–2)	1.5–4
Odontostyle length	8	8 (7–9)	7.5 (7–8)	8 ± 0.7 (7–11)	8 (7–9)	7–11
Odontophore length	9	9 (8–9)	9 (8–11)	9 ± 0.7 (6–10)	9 (8–10)	6–11
Guiding ring from anterior end	6	6 (5–6)	5 (5–5.5)	5 ± 0.3 (5–6)	5 (4–6)	4–6
Nerve ring from anterior end	81	79 (68–97)	74 (70.5–83.0)	72.0 ± 4.5 (65–80)	74 (70–79)	65–97
Pharyngeal length	193	184 (163–223)	175 (141–194)	172 ± 7.0 (162–187)	177 (164.5–189)	141–223
Expanded part of pharynx	78	75 (69–83)	66.5 (49–75)	70 ± 3.2 (62–77)	72 (63–80)	49–83
Cardia length	8	7 (5.0–10)	6.5 (5–9)	7 ± 1.1 (5–10)	8 (6–9)	5–10
Body diameter at neck base	23	24 (22–26)	21 (19–23)	21.0 ± 1.4 (17–24)	22 (20–24)	17–26
Body diameter at mid-body	23	24 (22.5–27)	22.0 (20–24)	22 ± 1.9 (19–26)	23 (21–25)	19–27
Body diameter at anus	16	17 (14–19)	16.5 (15–18)	18 ± 2.0 (14–22.0)	17 (16–18)	14–22.0
Anterior genital branch	71	87 (66.0–137)	73 (57.5–95)	78 ± 9.3 (56–95)	78 (57–87)	56–137
Posterior genital branch	76	84 (58–115)	68 (48–93)	75 ± 10.1 (57–102.0)	69 (63–75.5)	48–115
Vagina length	11	11 (10–12)	10 (8–11)	10.0 ± 0.7 (8–11)	11 (9–11)	8–12
Vulva from anterior end	415	421 (387–460)	415.5 (368–454)	399 ± 21.6 (353–440)	417 (394–443)	353–460
Prerectum length	40	51 (36–73)	57 (44–68)	42 ± 9.6 (25–58)	52 (35–64.5)	25–73
Rectum length	16	22.5 (20.0–24)	20 (17–23)	20 ± 2.8 (15–25)	23 (20.0–27)	15–27
Tail length	18	18 (16–21)	18.0 (16–20)	17.5 ± 1.3 (15–20)	18 (16–19.5)	15–21

**Notes.**

All measurements are in µm (except for ‘L in mm) and are given as mean (minimum- maximum) with SD indicated when *n* > 30.

n number of specimens observed L body length a L/maximum width b L/ pharyngeal L c L/tail length c′ tail length/ body diameter at anus V distance of vulva from anterior end × 100/L G1 anterior uterine sac × 100/L G2 posterior genital branch × 100/L

### Discussion on new genus classification

In Dorylaimida, there are two cuticle types: (a) dorylaimoid: inner layer not loose and without radial elements; (b) tylencholaimoid: inner layer loose with irregular radial elements. Among all the families, only Leptonchidae and Tylencholaimidae had tylencholaimoid cuticle, whereas the others had dorylaimoid cuticle. The morphology of the basal expansion of pharynx of the new genus is similar to *Tylencholaimus* of Tylencholamidae. However, *Paratylencholaimus* gen. nov. has dorylaimoid cuticle that is different from the tylencholaimoid cuticle of *Tylencholaimus*. Given the dorylaimoid cuticle, papiliform labial sensory organs, symmetrical odontostyle and odontophore and pharynx two parts, *Paratylencholaimus* gen. nov. is placed under the family Tylencholaimellidae, and it can be easily differentiated from the other genera of Tylencholaimellidae by having cylindrical basal expansion occupying one-third of the pharynx. Besides, the new genus showed a close relationship with the species of Tylencholaimellidae not *Tylencholaimus* spp. in both the 18S rDNA and D2–D3 region of 28S rDNA Bayesian trees. Thus, the present taxonomic status of the new genus is supported by both the morphological and phylogenetic results.

According to the latest classifications of Tylencholaimellidea (Peña Santiago, 2006; Peña Santiago, 2014), *Paratylencholaimus* gen. nov. should be placed under the subfamily Tylencholaimellinae due to its amphideal fovea that is not sclerotized. However, *Paratylencolaimus* gen. nov. and its close relative genus *Goferus* has odontophore without distinct basal knobs and basal expansion occupying greater than one-fifth of the total pharyngeal length. In contrast, the remainder genera of Tylencholaimellinae (*Dorella* Jairajpuri, 1964, *Margollus* Peña-Santiago, Peralta & Siddiqi, 1993, *Tylencholaimellus* Cobb in Cobb, 1915, *Doryllium* Cobb, 1920, *Oostenbrinkella* Jairajpuri, 1965 and *Phellonema* Thorne, 1964) except *Agmodorus* Thorne, 1964 (see further) have distinct basal knobs and basal expansion about or less than one-fifth of the total pharyngeal length. To adjust this, we propose to place the new genus and *Goferus* under a new subfamily, namely, Paratylencholaiminae subfam. nov.

**Table:** 

**Paratylencholaiminae subfam. nov.**
urn:lsid:zoobank.org:act:F5D7E807-6CF2-48CE-B771-D48B3594806D

### Diagnosis

Dorylaimida, Dorylaimina, Tylencholaimellidae. Cuticle dorylaimoid without radial refractive elements. Lip region continuous or offset from the body. Amphideal fovea not sclerotized. Odontostyle straight without pieces, odontophore without basal knobs. Expanded part of pharynx pyriform and unconstructed. Female genital system didelphic. Tail elongate to conoid-rounded. Two genera.

### Type genus

*Paratylencholaimus* gen. nov.

### Other genus

*Goferus* Jairajpuri & Ahmad, 1992

### Remarks

According to Peña Santiago (2006) and Peña Santiago (2014), Tylencholaimellidae includes two subfamilies: (a) Athernematinae Ahmad & Jairajpuri, 1978: amphidial fovea bilobed and strongly sclerotized, odontostyle asymmetrical and arcuate without accessory pieces, odontophore simple, pharyngeal expansion pyriform, female mono-opisthodelphic and tail filiform in both sexes; (b) Tylencholaimellinae Jairajpuri, 1964: amphidial fovea not sclerotized, odontostyle tubular, occasionally more attenuated, and with or without accessory pieces, odontophore with or without basal knobs, basal expansion occupying about one-fifth of the total pharyngeal length, female didelphic or monodelphic and tail long and filiform to short and hemispheroid. Detailed characteristics of the genera of Tylencholaimellidae were listed and compared in Table 4. And the classifications according to Peña Santiago (2006); Peña Santiago (2014) are as follow:

**Table:** 

Tylencholaimellidae
Athernematinae Ahmad & Jairajpuri, 1978
*Athernema* Ahmad & Jairajpuri, 1978
Tylencholaimellinae Jairajpuri, 1964
*Agmodorus* Thorne, 1964
*Dorella* Jairajpuri, 1964
*Doryllium* Cobb, 1920
*Goferus*Jairajpuri & Ahmad, 1992
*Margollus* Peña-Santiago, Peralta & Siddiqi, 1993
*Oostenbrinkella* Jairajpuri, 1965
*Phellonema* Thorne, 1964
*Tylencholaimellus* Cobb in Cobb, 1915

The odontostyle of *Athernema* and *Agmodorus* has not the typical tube shaped. We also found that these two genera with the arched odontophore, conoid to filiform tail and the opisthodelphic female genital system are more closely related to the family Mydonomidae Thorne, 1964 which is mainly characterized by having odontostyle asymmetry, odontophore straight or arched, basal expansion cylindrical and no longer than one-third of the pharynx length, female didelphic or opisthodelphic. Thus, we propose to transfer *Athernema* and *Agmodorus* into the family Mydonomidae Thorne, 1964 according to the morphology mentioned above, and under the subfamily Mydonominae Thorne, 1964 according to the body length less than three mm, and cancel the subfamily Athernematinae.

**Table 4:** Comparisons of some morphology of the genera of the family Tylencholaimellidae Jairajpuri, 194 and Mydonomidae Thorne, 1964 (classification sensu Peña Santiago (2014)).

Family	Subfamily	Genus	Amphid	Odontostyle	Odontophore	Basal expansion of pharynx	Genital system	Tail
Tylencholaimellidae	Athernematinae	*Athernema*	bilobed, sclerotized	arcuate	arcuate, not knobbed	1/5, not constricted	opisthodelphic	filiform
	Tylencholaimellinae	*Agmodorus*	goblet	short as if broken off at tip	arcuate, not knobbed	very short, pyfiform,constricted	opisthodelphic	elongate or clavate with long terminal hyaline portion
		*Doryllium*	goblet	short, tubular	knobbed or flange	pyfiform, most constricted	opisthodelphic	short and rounded
		*Goferus*	goblet	narrow, straight	simple, not knobbed	cylindrus, not constricted	amphidelphic	short, conoid-rounded
		*Oostenbrinkella*	goblet	attenuated	strongly knobbed	very short, not constricted	opisthodelphic	filiform
		*Phellonema*	goblet	short	with basal knobs	cylindrus, constricted	didelphic	short, anus subterminal
		*Dorella*	goblet	with short ventral stiffening piece	knobbed	short, constricted	mono-prodelphic	short, conoid-rounded
		*Margollus*	goblet	attenuated with dorsal stiffening piece	knobbed	cylindrical	opisthodelphic	convex-conoid to hemispherical
		*Tylencholaimellus*	goblet	short, tubular with dorsal accessory piece	with basal knobs	bulb like or pyriform	opisthodelphic	rounded or conoid with rounded tip
		*Paratylencholaimus* gen. nov.	goblet	straight with distinct lumen	simple, not knobbed	long, occupying 34–45%	didelphic	rounded to conoid-round
Mydonomidae	Calolaiminae	*Calolaimus*	goblet	irregularly	straight, sclerotized	cylindrical, up to one-third	didelphic	elongate-conoid to filiform
		*Timmus*	goblet	irregular in outline	simple, not knobbed	short, cylindroid or bulb-like	amphidelphic	filiform
	Mydonominae	*Dorylaimoides*	goblet	asymmetrical	arcuate or angular	cylindrical, one-fourth to one-third	amphidelphic or opisthodelphic	short and rounded to elongate or filiform
		*Morasia*	goblet	asymmertrical	arcuate	cylindroid, about one-third	amphidelphic	elongate-conoid in female, rounded in male
		*Mydonomus*	goblet	asymmertrical	arcuate	weak bulb, enclosed in muscular sheath	amphidelphic	short, bluntly conoid

The main characteristics of the families Mydonomidae, Tylencholaimellidae and the subfamily Tylencholaimellinae should be revised as follow:

Mydonomidae: cuticle dorylaimoid; lip region continuous or slightly offset, occasionally cap like; lips rounded, usually amalgamated; odontostyle short, asymmetry or not typical tubular, with distinct lumen; odontophore straight or arched; basal expansion cylindrical and no longer than one-third of the pharynx length, occasionally offset; female genital system didelphic-amphidelphic or mono-opisthodelphic; spicula dorylaimoid; ventromedial supplements spaced, 1–20; tail variable, short and rounded to elongate or filiform, similar or dissimilar in sexes.

Tylencholaimellidae: cuticle dorylaimoid; lip region cap-like, more or less offset, lips amalgamated; odontostyle short, tubular, occasionally with accessory stiffening piece; odontophore with or without basal knobs or flanges; basal expansion short pyriform, usually offset, occupying one-fifth to one-third of the pharynx length; female amphidelphic or opisthodelphic, exceptionally prodelphic; vulva transverse; spicula dorylaimoid; ventromedial supplements none to two, spaced; tail short and rounded to filiform, similar in sexes.

Tylencholaimellinae: amphideal fovea not sclerotized; odontostyle straight without accessory stiffening piece; odontophore with distinct basal knobs or flanges, basal expansion occupying about one-fifth of the total pharyngeal length, female didelphic or monodelphic and tail long and filiform to short and hemispheroid.

The new classifications of Mydonomidae and Tylencholaimellidae are:

**Table:** 

Mydonomidae Thorne, 1964
Calolaiminae Goseco, Ferris & Ferris, 1976
*Calolaimus* Timm, 1964
*Timmus* Goseco, Ferris & Ferris, 1976
Mydonominae Thorne, 1964
*Athernema* Ahmad & Jairajpuri, 1978
*Agmodorus* Thorne, 1964
*Dorylaimoides* Thorne & Swanger, 1936
*Morasia* Baqri & Jairajpuri, 1969
*Mydonomus* Thorne, 1964
Tylencholaimellidae Jairajpuri, 1964
Paratylencholaiminae subfam. nov.
*Goferus*Jairajpuri & Ahmad, 1992
*Paratylencholaimus* gen. nov.
Tylencholaimellinae Jairajpuri, 1964*Doryllium* Cobb, 1920
*Dorella* Jairajpuri, 1964
*Doryllium* Cobb, 1920
*Margollus* Peña-Santiago, Peralta & Siddiqi, 1993
*Oostenbrinkella* Jairajpuri, 1965
*Phellonema* Thorne, 1964
*Tylencholaimellus* Cobb in Cobb, 1915

### Key to the genera of Mydonomidae

**Table:** 

1	Body length 3–7 mm	2
	Body length under three mm	3
2	Adcloacal supplements two pairs	*Timmus* Goseco, Ferris & Ferris, 1976
	Adcloacal supplements one pair	*Calolaimus* Timm, 1964
3	Amphidial fovea bilobed, sclerotized	*Athernema* Ahmad & Jairajpuri, 1978
	Amhpidial fovea simple, not sclerotized	4
4	Odontostyle very short with apparently broken tip	*Agmodorus* Thorne, 1964
	Odontostyle normal	5
5	Tails dissimilar in sexes	*Morasia* Baqri & Jairajpuri, 1969
	Tails similar in sexes	6
6	Basal expansion of pharynx surrounded by a muscle sheath	*Mydonomus* Thorne, 1964
	Basal expansion of pharynx without muscle sheath	*Dorylaimoides* Thorne & Swanger, 1936

### Key to the genera of Tylencholaimellidae

**Table:** 

1	Odontostyle with a convex stiffening piece	2
	Odontostyle without stiffening piece	4
2	Stiffening piece ventral; ovary prevulval	*Dorella* Jairajpuri, 1964
	Stiffening piece dorsal; ovary postvulval	3
3	Labial framework sclerotized	*Margollus* Peña-Santiago, Peralta & Siddiqi, 1993
	Labial framework not sclerotized	*Tylencholaimellus* Cobb in Cobb, 1915
4	Odontophore simple without basal knobs	5
	Odontophore with distinct basal knobs	6
5	Lip region offset; basal part of pharynx cylindrical, expanded at posterior one-third	*Paratylencholaimus* gen. nov.
	Lip region continuous; basal part of pharynx pyriform, much shorter	*Goferus*Jairajpuri & Ahmad, 1992
6	Female didelphic-amphidelphic	*Phellonema* Thorne, 1964
	Female monodelphic-opisthodelphic	7
7	Tail filiform	*Oostenbrinkella* Jairajpuri, 1965
	Tail short, rounded	*Doryllium* Cobb, 1920

## Conclusions

Both the morphology and phylogenetic analysis results support that the three new species and the new genus are valid. The classifications and the main characteristics of the families Tylencholaimellidae and Mydonomidae are revised due to the propositions of Paratylencholaiminae subfam. nov. and *Paratylencholaimus* gen. nov. In the new classifications, *Athernema* and *Agmodorus* of Tylencholaimellidae are transferred into Mydonomidae, and the subfamily Athernematinae of Tylencholaimellidae was canceled. Keys to the genera of these two families are also provided. However, deeper inner relationships of the genera of Tylencholaimellidae and Mydonomidae remain unclear and more information including morphology and phylogeny of these two families are needed.

##  Supplemental Information

10.7717/peerj.7541/supp-1 File S1 Raw data of phylogenetic analysis based on the 18S rDNA

10.7717/peerj.7541/supp-2 File S2 Raw data of phylogenetic analysis based on the D2–D3 region of the 28S rDNA

## Additional Information and Declarations

### DNA Deposition

The following information was supplied regarding the deposition of DNA sequences: The sequences described here are accessible via GenBank: MG921272–MG921309, KU662324 and KU662325.

### Data Availability

The following information was supplied regarding data availability: The raw data for the phylogenetic analysis based on the 18S rDNA and the D2–D3 region of the 28S rDNA are available in the Supplemental Files.

### New Species Registration

The following information was supplied regarding the registration of a newly described species: Publication LSID: urn:lsid:zoobank.org:pub:427B5E52-23B0-4474-B179-4FA9FC5E7C9C. *Tylencholaimus zhongshanensis* sp. nov.: urn:lsid:zoobank.org:act:AFB00C8F-918E-422C-ABB6-71F4DFBCA3D5. *Dorylaimoides shapotouensis* sp. nov.: urn:lsid:zoobank.org:act:F6CB7E8A-0C6D-496F-9B98-2E151328E64F. *Paratylencholaiminae* n. subfam.: urn:lsid:zoobank.org:act:F5D7E807-6CF2-48CE-B771-D48B3594806D. *Paratylencholaimus* gen. nov.: urn:lsid:zoobank.org:act:4BFCC48B-38E2-449C-B1F5-338E46E7B099. *Paratylencholaimus sanshaensis* sp. nov.: urn:lsid:zoobank.org:act:63A581ED-4755-481F-B7A9-F71C79946A9C.
